# Supplementary material for: Splice-Junction-Based Mapping of Alternative Isoforms in the Human Proteome
Source: Cell Rep. Author manuscript; Available in PMC 2020 Jan 15. (PMC6961840; doi:10.1016/j.celrep.2019.11.026)
Supplement: 2 [file NIHMS1546469-supplement-2.zip › DF1/PXD010154/Heart/Heart_25_TTN_RVEAEPAEEVTIMEEKER.pdf]

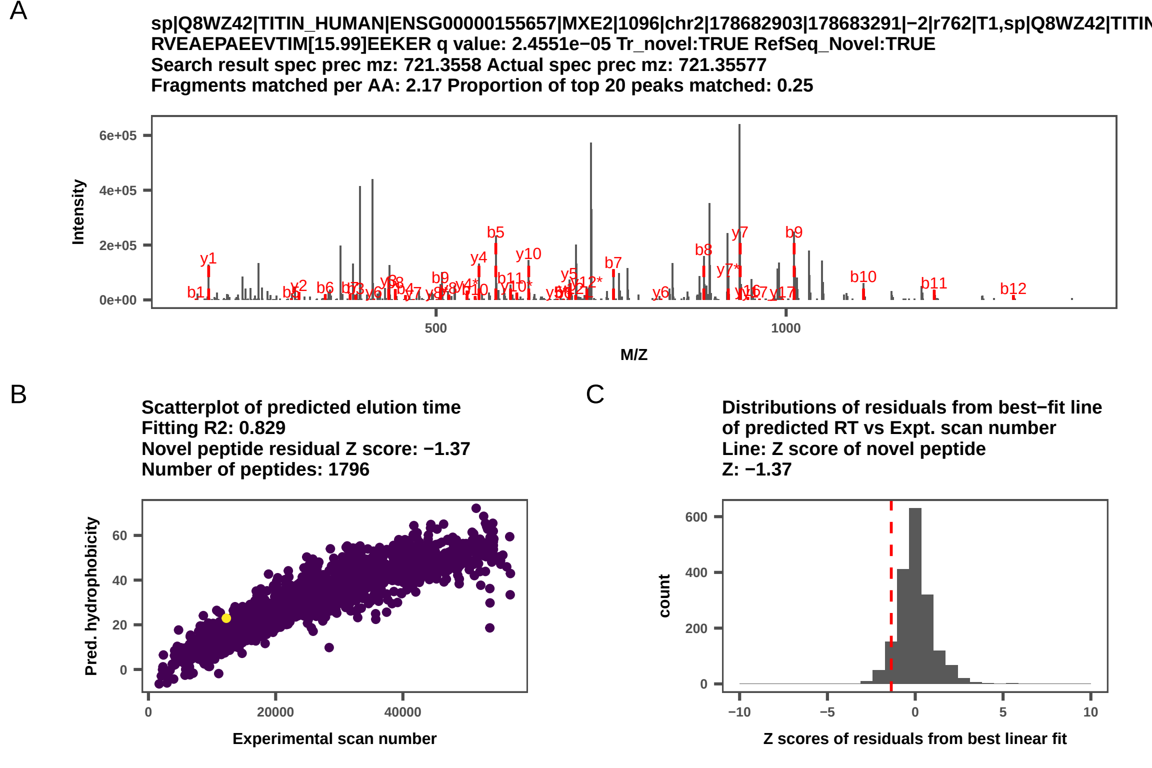
A
sp|Q8WZ42|TITIN_HUMAN|ENSG00000155657|MXE2|1096|chr2|178682903|178683291|−2|r762|T1,sp|Q8WZ42|TITIN
RVEAEPAEEVTIM[15.99]EEKER q value: 2.4551e−05 Tr_novel:TRUE RefSeq_Novel:TRUE
Search result spec prec mz: 721.3558 Actual spec prec mz: 721.35577
Fragments matched per AA: 2.17 Proportion of top 20 peaks matched: 0.25
Intensity
6e+05
4e+05
2e+05
0e+00
M/Z
500
1000
y1
b1
y2
b6
b5
y4
y10
b7
b8
y7
y7*
b9
b10
b11
b12
y6
B
Scatterplot of predicted elution time
Fitting R2: 0.829
Novel peptide residual Z score: −1.37
Number of peptides: 1796
Pred. hydrophobicity
60
40
20
0
0
20000
40000
Experimental scan number
C
Distributions of residuals from best−fit line
of predicted RT vs Expt. scan number
Line: Z score of novel peptide
Z: −1.37
count
600
400
200
0
−10
−5
0
5
10
Z scores of residuals from best linear fit
